# Primary Mesenteric Sertoli-Leydig Cell Tumor: A Case Report and Review of the Literature

**DOI:** 10.1155/2008/619637

**Published:** 2008-10-30

**Authors:** Amel Trabelsi, Soumaya Ben Abdelkarim, Mohamed Hadfi, Ridha Fatnaci, Wided Stita, Badreddine Sriha, Sadok Korbi

**Affiliations:** Department of Pathology, Farhat Hached Hospital, Sousse University, 4000 Sousse, Tunisia

## Abstract

The occurrence of primary sex cord-stromal tumors at extraovarian sites is exceedingly rare. We report a new case of Sertoli-Leydig cell tumor in the mesentery of a 78-year-old woman who presented with occlusive syndrome and reviewed the previously reported cases of extraovarian sex cord-stromal tumors in the English literature.

## 1. Introduction

The occurrence of primary sex cord-stromal tumors at extraovarian sites is extremely rare [1]. These tumors are predominantly granulosa cell tumors [1]. To our knowledge, this is the first case of primary mesenteric Sertoli-Leydig cell tumor.

## 2. Case report

**Figure 1:**
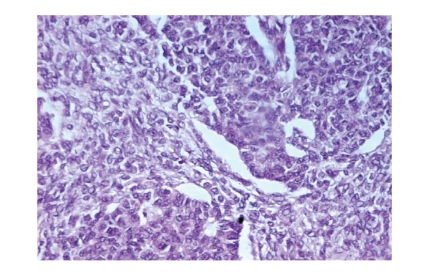
Cellular lobules and nests with poorly developed tubules (HEx200).

**Figure 2:**
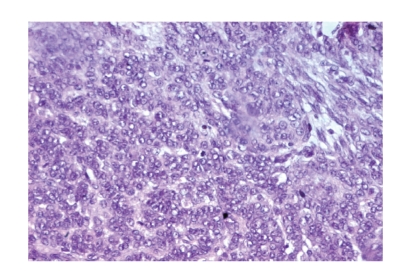
Sertoli cells with moderate atypia and mitotic figures (HEx200).

**Figure 3:**
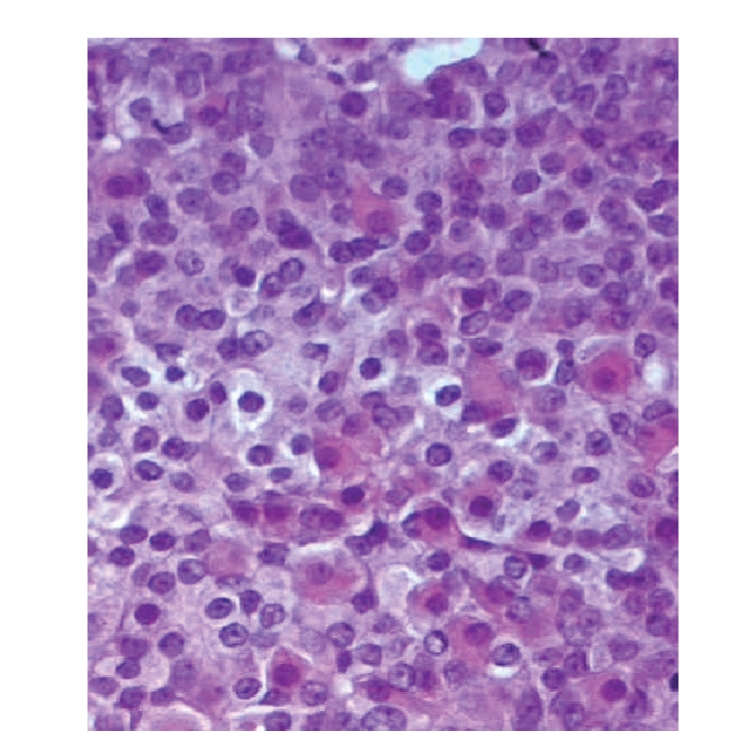
Leydig cells surrounded tumor lobules (HEx400).

**Figure 4:**
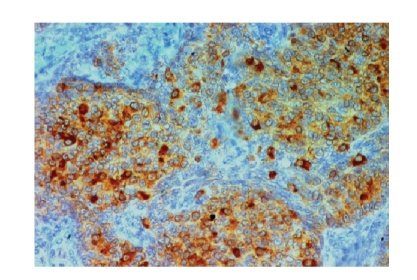
Tumor cells immunoreactive to anti-inhibin (IHCx400).

 A new case of a 78-year-old woman who presented with bowel obstruction is reported. Three years previously, she underwent bilateral salpingo-oophorectomy and total abdominal hysterectomy for bilateral mucinous cystadenoma and vaginal prolapse. Ultrasonography showed a solid mesenteric tumor; exploratory laparotomy showed a nodular solid tumor at the mesenteric border of the distal ileum, measuring 11 × 8  cm. Twenty centimeters of ileum containing the isolated lesion was resected. Macroscopically, the tumor was nodular and well circumscribed measuring 11 × 8  × 4 cm. The cut surface was composed of pale yellow-grey soft tissue with foci of haemorrhage. The tumor was limited to mesentery without extension to the ileal wall. Microscopic examination revealed cellular lobules with nests and poorly developed tubules of Sertoli cells (Figure 1) that showed moderate atypia and mitotic figures that average 5–10 per high-power field (Figure 2). Leydig cells are found at the periphery of the cellular lobules (Figure 3). By immunohistochemistry, the neoplastic cells showed positive staining for antibodies against inhibin (Figure 4) and vimentin. Tumor cells were negative for EMA (epithelial membrane antigen), calretinin, synaptophysin, chromogranin A, and CD117 (c-kit). The diagnosis of Sertoli-Leydig cell tumor with intermediate differentiation of the mesentery was established. The patient has not received any additional therapy, yet she remains free of disease after five years.

## 3. Discussion

The occurrence of primary sex cord-stromal tumor at extraovarian sites is extremely rare [1], such that in the English literature only 13 cases have been reported [1–13]. Eight were classified as granulosa cell tumors [1–6, 11, 12], two as thecomas [7, 8], two as sex cord-stromal tumors, and one as an unclassified form of stromal sex cord tumor [13]. The sites of origin were usually within the pelvis [1]: six arose in the broad ligament [1], two in the retroperitoneum [2, 12, 13], one in the fallopian tube [10], one in an umbilical herniae sac [9], one in the adrenal gland [6], and one in the pelvic sidewall [11]. These previous reported cases are summarized in Table 1.

To our knowledge, this is the first case of a primary mesenteric Sertoli-Leydig cell tumor. Clinically, these tumors sometimes produce estrogen [1, 6]. However, in our case, no biologic assay for estrogen content of the patient's urine or blood was performed, because the nature of the tumor was not suspected until it was removed.

The histogenesis of extraovarian sex cord-stromal tumors has been reviewed in the literature [12].

In recent years, several investigators have claimed that the sex cords may originate from the mesonephros [14]. A dualistic theory of both coelomic epithelium and mesonephros in the origin of the pregranulosa cells has also been proposed [12]. Accordingly, the mesonephros itself or its functional influence seems to be necessary for creating the sex cords. This is consistent with gonad formation being limited to the gonadal ridge and may explain why the sites of origin of extraovarian sex cord-stromal tumors are limited to the broad ligament, retroperitoneum, mesentery, and adrenal gland, all of which differentiate close to the mesonephros and mesonephric duct [1]. Sex cord-stromal tumors can be difficult to distinguish from several other neoplasms including undifferentiated carcinoma, gastrointestinal stromal tumors (GIST) or metastatic melanoma. The immunohistochemical findings are invaluable in confirming the diagnosis.

The prognosis for extraovarian sex cord-stromal tumors seems to be favorable; however, reported cases and clinical experiences are limited.

## Figures and Tables

**Table 1:** Reported cases of extraovarian sex cord-stromal tumors.

Reference	Age (years)	Histological diagnosis	Site of origin	Recurrence
[2]	51	Granulosa cell tumor	Retroperitoneum	No
[3]	37	Granulosa cell tumor	Broad ligament	No
[4]	30	Granulosa cell tumor	Broad ligament	No
[5]	40	Granulosa cell tumor	Broad ligament	No
[6]	52	Granulosa cell tumor	Adrenal gland	No
[7]	76	Fibrothecoma	Broad ligament	No
[8]	70	Thecoma	Broad ligament	No
[9]	66	Sex cord tumor with annular tubes	Umbilical herniae sac	No
[10]	32	Sex cord tumor with annular tubes	Fallopian tube	No
[1]	45	Granulosa cell tumor	Broad ligament	Yes
[11]	67	Granulosa cell tumor	Pelvis	No
[12]	54	Granulosa cell tumor	Retroperitoneum	No
[13]	32	Unclassified stromal sex cord tumors	Retroperitoneum	No

Our case	78	Sertoli-Leydig cell tumor	Mesentery	No
